# The effects of acylation stimulating protein supplementation *VS *antibody neutralization on energy expenditure in wildtype mice

**DOI:** 10.1186/1472-6793-10-4

**Published:** 2010-04-23

**Authors:** Sabina Paglialunga, Alexandre Fisette, Mercedes Munkonda, Ying Gao, Denis Richard, Katherine Cianflone

**Affiliations:** 1 Centre de Recherche de l'Institut Universitaire de Cardiologie et de Pneumologie de Québec, Université Laval, Québec, QC, G1V 4G5, Canada; 2 Biochemistry Department, McGill University, Montreal, QC, H3G 1Y6, Canada; 3 Department of Cardiology, The First Affiliated Hospital, Xinjiang Medical University, Xinjiang Uygur Autonomous Region, China

## Abstract

**Background:**

Acylation stimulating protein (ASP) is an adipogenic hormone that stimulates triglyceride (TG) synthesis and glucose transport in adipocytes. Previous studies have shown that ASP-deficient C3 knockout mice are hyperphagic yet lean, as they display increased oxygen consumption and fatty acid oxidation compared to wildtype mice. In the present study, antibodies against ASP (Anti-ASP) and human recombinant ASP (rASP) were tested *in vitro* and* in vivo*. Continuous administration for 4 weeks via osmotic mini-pump of Anti-ASP or rASP was evaluated in wildtype mice on a high-fat diet (HFD) to examine their effects on body weight, food intake and energy expenditure.

**Results:**

In mature murine adipocytes, rASP significantly stimulated fatty acid uptake (+243% vs PBS, P < 0.05) while Anti-ASP neutralized the rASP response. Mice treated with Anti-ASP showed elevated energy expenditure (P < 0.0001), increased skeletal muscle glucose oxidation (+141%, P < 0.001), reduced liver glycogen (-34%, P < 0.05) and glucose-6-phosphate content (-64%, P = 0.08) compared to control mice. There was no change in body weight, food intake, fasting insulin, adiponectin, CRP or TG levels compared to controls. Interestingly, HFD mice treated with rASP showed the opposite phenotype with reduced energy expenditure (P < 0.0001) and increased body weight (P < 0.05), cumulative food intake (P < 0.0001) and liver glycogen content (+59%, P < 0.05). Again, there was no change in circulating insulin, adiponectin, CRP or TG levels, however, plasma free fatty acids were reduced (-48%, P < 0.05).

**Conclusion:**

*In vitro*, Anti-ASP effectively neutralized ASP stimulated fatty acid uptake. *In vivo*, Anti-ASP treatment increased whole body energy utilization while rASP increased energy storage. Therefore, ASP is a potent anabolic hormone that may also be a mediator of energy expenditure.

## Background

Acylation stimulating protein (ASP) is a circulating adipokine elevated in obesity, diabetes and cardiovascular disease [1] as well as other metabolic disorders with hyperlipidemia such as polycystic ovarian syndrome (PCOS) [2], hypothyroidism [3] and lipoprotein lipase (LPL) deficiency [4]. ASP influences fat storage by stimulating diacylglycerol acyltransferase (DGAT) activity, the rate limiting step in triglyceride (TG) synthesis [5], increasing glucose transporter GLUT4 translocation [6], indirectly stimulating LPL activity in adipose tissue [7], and inhibiting lipolysis [8]. These effects are mediated through the C5L2 receptor, a G protein-coupled receptor highly expressed in adipose tissue depots, skeletal muscle and liver [9,10].

ASP (C3adesArg) is the cleavage product of complement C3 (reviewed in [1]); accordingly C3 knockout (C3KO) mice are ASP-deficient. Furthermore, C3KO mice are characterized by having increased food intake [11,12], delayed postprandial dietary triglyceride clearance [13,14], increased oxygen consumption [12,15], and greater muscle fatty acid oxidation relative to glycolysis [12]. ASP receptor KO mice, C5L2KO, share a similar phenotype with C3KO mice. Overall fatty acid oxidation is augmented (reduced respiratory quotient), food intake is increased, and they also display delayed postprandial lipid clearance [16]. It is interesting to note that mice lacking either ASP or C5L2 from *in utero *have less TG storage capacity in adipose tissue and greater fatty acid oxidation in muscle. Moreover, these effects have also been observed in wildtype (WT) mice treated acutely with ASP/C5L2-neutralizing antibodies [17].

In a previous short-term pilot study, antibodies designed to block ASP-C5L2 interaction, i.e., antibodies against ASP (Anti-ASP) or C5L2 (Anti-C5L2), were both shown to inhibit ASP stimulated TG synthesis and glucose transport in preadipocytes and mature adipocytes [17]. These antibodies were then administered daily to WT mice on a low-fat diet over the course of a short 10 day study. The results showed no difference in body weight or food intake with antibody treatment, however there were significant changes in lipid metabolism and adipose tissue and skeletal muscle enzyme activity. Adipose tissue TG mass and LPL activity were reduced, while skeletal muscle AMPK activity was increased [17]. Therefore, even with short term administration, neutralizing antibodies blocking ASP-C5L2 interaction resulted in beneficial altered lipid distribution and energy utilization in WT mice.

Based on the previous observations of altered muscle activity and substrate utilization, the present study examines energy expenditure and substrate storage over a longer timeframe (4 weeks) in WT mice administered a constant delivery of antibody. Further, the mice were challenged with a high-fat diet two weeks prior to, and during, the treatment. In addition, in the present study, we investigated the consequences of ASP supplementation in WT mice following initiation of a high-fat diet.

## Methods

### Anti-ASP and recombinant ASP production

Polyclonal rabbit antibody against human ASP (Anti-ASP) was developed as previously described [18]. The IgG fraction was isolated from plasma by gamma sepharose chromatography (GE Healthcare, Chicago, IL, USA) and the polyclonal antibody cross-reacted with mouse ASP [17]. Non-immune IgG (NI-IgG) was purchased from Sigma (Sigma, St Louis, MO, USA). For the production of recombinant ASP (rASP), the portion of the human C3 gene representing ASP (C3adesArg) was cloned into the NcoI/EcoRI restriction sites of pET32a(+) (Novagen, Madison, WI, USA). An extra alanine (GCC) codon was incorporated to be able to use NcoI as a cloning site. The vector was digested with NdeI to excise out the thioredoxin tag (TRX.tag) and religated to obtain pET_his_ASP vector containing the His-tagged ASP sequence. The cell line used to produce recombinant ASP was Origami B (DE3) (Novagen), a BL21 derived cell line lacking thioredoxin and glutathione reductase genes. Frozen OriB-pET-his-ASP scrapes were inoculated and grown overnight at 37°C with shaking (220 rpm) in bacterial media (25 g/L LB Broth, EMD Biosciences, Madison, WI, USA) supplemented with 10 ug/mL kanamycin (Invitrogen, Burlington, ON, Canada) and 100 ug/mL carbenicillin (Invitrogen). Cultures were diluted (1.5 mL/L) in bacterial media, incubated 5-8 hours (37°C, 220 rpm) until an OD_600 _of 0.6-0.8 was reached, then supplemented with IPTG (Isopropyl ß-D-1-thiogalactopyranoside, QIAGEN, Mississauga, ON, Canada, 1 mM final concentration) and incubated overnight (25°C, 220 rpm). Cell pellets were collected (centrifugation 3000 ×g, 20 min), lysed with 10 mL Bugbuster (EMD Biosciences), 10 mg lysozyme (Sigma) and 10 μL benzonase nuclease (20 U/mL, EMD Biosciences), vortexed well, shaken 30 min at RT, centrifuged (3000 g, 5 min, 4°C) and the supernatant decanted. The lysis step was repeated with an additional 10 mL, then 5 mL. All supernatants were pooled, acidifed with 10N HCl (final 1 N HCl), stirred (15 min RT), centrifuged (3000 g, 20 min, 4°C), pooled, adjusted to pH 7.4 with 10 N NaOH and centrifuged (3000 g, 20 min, 4°C). rHis-ASP was purified with binding to Ni^2+^-Sepharose column (GE Healthcare). Following loading of rHis-ASP, the column was washed with buffer (50 mM NaH_2_PO_4_, 300 mM NaCl, 10 mM imidazole, pH 74.) and eluted with acidified buffer (0.625 N HCl in buffer, pH ~2.0). For removal of rHis-tag, rHis-ASP solution was carefully adjusted to pH 8.0, enterokinase (New England Biolabs, Pickering, ON, Canada) was added (0.063 ug/20 mL), and incubated overnight at RT (18°C) with gentle shaking. rASP was purified by HPLC as previously described [19].

### In vitro fatty acid uptake

3T3-L1 preadipocytes cells were obtained from ATCC (Manassas, VA, USA). Cells were routinely grown in Dulbecco's modified Eagle's medium (DMEM) + 10% (v/v) fetal calf serum (Invitrogen) at 37°C, 5% CO_2_. 3T3-L1 preadipocytes were differentiated into mature adipocytes as described previously [7]. Differentiated adipocytes in 48 well plates were preincubated (37°C, 5% CO_2_) in serum free medium for 1 hour then incubated with PBS (baseline synthesis), insulin (100 nM) (Sigma), rASP (100 nM), or rASP (100 nM) + Anti-ASP (0.2, 0.3, 0.4 μ g/mL, added before the addition of ASP) for 1 hour. Uptake of fluorescently labeled fatty acids was measured over time in adipocytes as described [20,21] using a QBT™ fatty acid uptake assay kit (Molecular Devices, Sunnyvale, CA, USA) as outlined by the manufacturer. Following the addition of BODIPY-fatty acid, fatty acid uptake is measured in real-time over 120 min in a bottom-reading fluorescent microplate reader.

### Mice

Male C57BL/6 mice (aged 7-8 weeks) were purchased from Charles River Laboratories (Wilmington, PA, USA). The mice were maintained on a high-fat diet (45% kcal from fat, Research Diet, New Brunswick, NJ, USA) throughout the study (total of 6 weeks) and housed individually in a sterile barrier facility with a 12 h light: 12 h dark cycle. Two weeks following the start of the high-fat diet regimen, the mice were surgically implanted with an osmotic mini-pump (model 2004) (Durect Corp., Cupertino, CA, USA). Briefly, the mice were first sedated with ketopiofen (5 mg/kg) then anesthetized with isofluorane. Under sterile conditions, a small incision was made between the skin and scapulae and the osmotic pump was inserted subcutaneously into the mouse. The incision was then closed with sutures according to manufacturer's instructions. All protocols were conducted in accordance with the CACC guidelines and approved by the University Animal Care Committee. The mice were divided into four groups where they were continuously delivered; i) non-immune IgG n = 6 (2 μg/μL); ii) Anti-ASP n = 7 (4 μg/μL); iii) PBS (vehicle) n = 6; or iv) recombinant ASP (95 pmol/μL) in PBS n = 5 for 4 weeks at a flow rate of 0.25 μL/hr. Twenty-eight days following mini-pump insertion (6 weeks on high-fat diet), mice were fasted overnight, euthanized, and tissues, blood samples and the mini-osmotic pumps were collected. Average pump rate was calculated as the difference between the initial volume loaded and the residual volume divided by 28 days.

### Plasma Measurements

Overnight fasting plasma samples were taken 9 days after the surgery by submandibular bleed and during sacrifice by cardiac puncture (day 28). Plasma human rASP levels were measured by an in-house ELISA which does not cross-react with mouse ASP, background non-specific (PBS) values were subtracted [18]. Rabbit IgG was measured by Easy-titer IgG assay kit (Thermo Fisher Scientific Inc., Rockford, IL, USA), to track the efficacy of pump delivery. Plasma TG (Roche Diagnostics, Indianapolis, IN, USA), non-esterified fatty acids (NEFA) (Wako Chemicals, Richmond, VA, USA) and glucose (Sigma) were measured using colorimetric enzymatic kits. Insulin and adiponectin were measured using RIA kits (Linco, St.Charles, MO, USA); interleukin-6 (IL-6) (BD, Franklin Lakes, NJ, USA) and CRP (Immunology, Consultants Laboratory, Inc., Newberg, OR, USA) were measured by ELISA. C3 was measured by immunoturbidimetric assay (Kamiya Biomedical Company, Seattle, WA, USA).

### Body Weight, Food Intake and Indirect Calorimetry

Body weight and food intake were measured 3 times a week. Food intake results are reported as cumulative food intake in kcal. Food efficiency was calculated as the food intake in kcal over body weight gain in grams. Calorimetry measurements were taken 15-17 days after the surgery. Oxygen consumption (VO_2_) and carbon dioxide production (VCO_2_) were measured over a 24 hour period in an open circuit system after 48 hours equilibration as previously described [22]. VO_2 _and VCO_2 _were calculated as mL/kg/min and RQ (respiratory quotient) was taken as the quotient of VCO_2_/VO_2_. Energy expenditure (kcal/kg/min) was calculated as 3.91VO_2_+1.1VCO_2_[23].

### Skeletal muscle ex vivo assays

Quadriceps muscles were excised and placed into room temperature Krebs-Ringer Buffer (KRB) containing 1% fatty acid free BSA and 5 mM glucose. For fatty acid oxidation, the muscle was cut into small pieces then incubated with 1 mM palmitic acid [^3^H palmitate, 5 μCi/μL] (Perkin-Elmer Life Sciences, Boston, MA, USA) in KRB for 2 hours. Oxidation was measured as the production of ^3^H_2_O into the aqueous phase following a Folch extraction as previously described [16]. The organic extract was dried down in a speedvac, resuspended in 100 μL chloroform/methanol (2:1) and lipids were separated using silica thin layer chromatography plates. The extracted lipids were separated using heptane/isopropyl ether/acetic acid (60:40:4) as the separation solvent for 45 min. The plate was developed in iodine vapour and diglyceride and triglyceride bands were identified and scraped into scintillation vials for counting. Following overnight solubilization of the tissue remnants in NaOH, total proteins were measured by Bradford assay (BioRad, Hercules, CA, USA). Glucose oxidation was performed as previously described [12].

### Substrate content and enzyme activity assays

Liver glycogen was degraded into glycosyl units by incubating small liver pieces (~50 mg) in 1 M HCl at 100°C for 3 hours [24]. The extracts were neutralized with TRIS-KOH and the supernatant was assayed for glucose using a commercial colorimetric kit (Sigma). Results are expressed as μmoles/g glycosyl units per liver weight. For glucose-6-phosphate measurements, small liver pieces (~25 mg) were homogenized in 6% perchloric acid. The supernatant was clarified by centrifugation and neutralized with KOH in the presence of phenol red. The resulting potassium perchlorate precipitate was removed by centrifugation and the supernatant was enzymatically assayed for glucose 6-phosphate by following NADPH accumulation at 340 nm in presence of glucose 6-phosphate dehydrogenase. Results are expressed as nmoles/g liver sample. Neutral lipids were extracted from muscle and liver samples (30-50 mg) in a heptane/isopropanol (3:2) solution overnight at 4°C. The next day organic extracts were assessed for TG content using a commercial colorimetric kit (Roche Diagnostics) and total protein content was measured as described above. Results are expressed as μmoles of TG per gram of protein. Glucokinase (GK) enzyme activity was measured as previously described [12] however; solution volumes were modified for a 96-well plate and ODs were measured on a plate reader (BioTek Instruments, Inc. Winooski, VT, USA).

### Statistics

Results are expressed as means ± SEM. Results were analyzed by two-way ANOVA (two variables), one-way ANOVA followed by Dunnett's post-hoc test (one variable, more than two groups) or two-tail t-test (two groups). For indirect calorimetry measurements body weight and food intake, Group and Time P values are indicated in the graphs. Average area-under the curve (AUC) was calculated for light (12 hours) and dark (12 hour) energy expenditure. Significance was set as P value < 0.05, where NS represents non-significant values. All graphs and statistical analyses were performed using GraphPad Prism 5.0 (GraphPad Software, San Diego, CA, USA).

## Results

### Anti-ASP blocks in vitro ASP-stimulated fatty acid uptake

**Figure 1:**
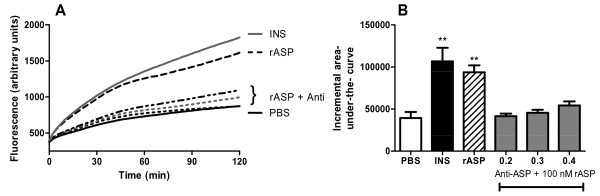
***In vitro *fatty acid uptake**. (A) Fluorescent BODIPY fatty acid uptake in real-time analysis over 120 mins. Differentiated adipocytes were treated with PBS (solid black line), insulin (solid grey line, 100 nM), rASP (hatched black line, 100 nM) or rASP (100 nM) + Anti-ASP, at the following concentrations from bottom to top: 0.2, 0.3 and 0.4 μg/mL, for n = 3. (B) Incremental area-under-the-curve (AUC) analysis of PBS (white solid bar) insulin (black solid bar, 100 nM), rASP (white hatched bar, 100 nM) and rASP (100 nM) + Anti-ASP (gray solid bars, concentrations as indicated) for n = 3. All values are presented as mean ± SEM, where ** P < 0.01.

Recombinant ASP (rASP) activation and antibody neutralization were first tested in an *in vitro *system. Insulin (positive control, a known lipogenic hormone) and rASP significantly stimulated fatty acid uptake in murine adipocytes to a similar degree (Figure 1A), with maximal stimulation of 285 ± 49% (P < 0.01) for insulin and 243% ± 24% (P < 0.05) for rASP, vs PBS set at 100%. This is also presented as a 2.7-fold (P < 0.01) increase for insulin and 2.4-fold (P < 0.01) increase for rASP in the incremental area-under-the-curve (Figure 1B). The addition of increasing concentrations of Anti-ASP significantly reduced rASP stimulated fatty acid uptake to basal levels (maximal stimulation: 98% ± 4%, 120% ± 10%, and 144% ± 18%, vs PBS set at 100% for 0.2, 0.3 and 0.4 μg/mL Anti-ASP respectively, not significantly different from PBS) (Figure 1A and 1B).

### Monitoring exogenous antibody levels and plasma hormones

**Figure 2:**
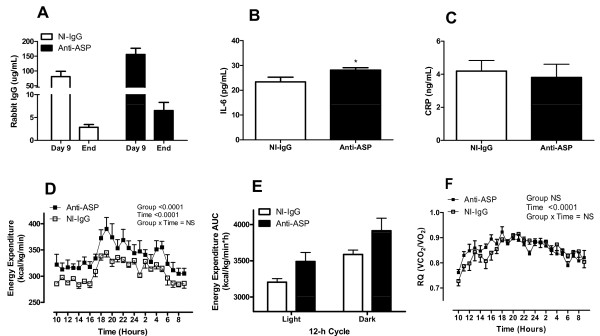
**Plasma antibody, IL-6, CRP and indirect calorimetry results for mice treated with ASP-neutralizing antibody or control antibody**. Fasting plasma samples from NI-IgG treated mice (n = 6, white bars) and Anti-ASP treated mice (n = 7, black bars). (A) Polyclonal rabbit antibody detected in mouse plasma following 9 days and 4 weeks treatment. (B) Plasma IL-6 and (C) CRP levels at end of treatment. Results were analyzed by unpaired two-tailed t-test. (D) Energy expenditure measured over 24 hours for NI-IgG treated mice (n = 6, white squares) and Anti-ASP treated mice (n = 7, black squares). Results were analyzed by two-way ANOVA. Group and time P values are reported in graph, their interaction was not significant. Analysis of light and dark periods separately (2-way ANOVA) indicated a significant difference between Anti-ASP and NI-IgG for both time periods (P < 0.0001) (E) Light and dark phase energy expenditure calculated as the area-under the curve (AUC). (F) respiratory quotient (RQ) measured over 24 hours for NI-IgG treated mice (white squares) and Anti-ASP treated mice (black squares). Results were analyzed by two-way ANOVA. Group and time P values are reported in graph, their interaction was not significant. All values are presented as mean ± SEM, where * P < 0.05.

Mice were treated by osmotic mini-pump for 4 weeks with neutralizing Anti-ASP or control non-immune IgG (NI-IgG). Plasma samples were taken 9 days after implantation of the osmotic pump and at the end of the study to track the amount of antibody delivered into circulation. Figure 2A shows that on day 9 rabbit NI-IgG and Anti-ASP antibodies were detected (~4% of starting material). However, by the end, the levels of both NI-IgG and Anti-ASP were greatly reduced (~0.1% of starting material). Since there was no difference in osmotic mini-pump rate for PBS, rabbit antibodies or rASP treatments (data not shown), reduced antibody levels may be attributed to enhanced antibody clearance. Anti-ASP blocking antibody treatment had no effect on mouse body weight (NI-IgG: 29.2 ± 0.4 g and Anti-ASP: 29.6 ± 0.3 g, NS, n = 6-7), average food intake (NI-IgG: 13.8 ± 0.5 kcal/day and Anti-ASP: 14.3 ± 0.5 kcal/day, NS, n = 6-7) or food efficiency (NI-IgG: 200.1 ± 21.2 kcal/g and Anti-ASP: 165.6 ± 25.8 kcal/g, NS, n = 6-7), consistent with our previous report during a 10 day study [17]. There was also no difference in adipose tissue depots, liver or spleen weight between the two groups (data not shown). IL-6 levels were slightly but significantly elevated in Anti-ASP mice (P < 0.05, Figure 2B). However, CRP levels, a marker of inflammation, were normal (Figure 2C). Anti-ASP did not have an effect on fasting glucose levels after nine days of treatment (NI-IgG: 6.22 ± 0.62 mmol/L and Anti-ASP: 6.58 ± 0.54 mmol/L, NS, n = 6-7). Furthermore, there was no difference in plasma adiponectin, insulin, C3, or lipid levels between the two groups at the end of the study (Table 1).

**Table 1:** NI-IgG and Anti-ASP Treated Mice Hormone and Lipids Levels

Plasma	NI-IgG (n = 5)	Anti-ASP (n = 6)
Adiponectin (ug/mL)	7.67 ± 0.51	8.85 ± 0.71

Insulin (ng/mL)	0.30 ± 0.07	0.28 ± 0.03

C3 (mg/mL)	0.41 ± 0.02	0.37 ± 0.03

Triglycerides (mmol/L)	0.65 ± 0.02	0.56 ± 0.08

NEFA (mmol/L)	0.99 ± 0.22	0.57 ± 0.05

Values are presented as mean ± SEM. No statistically significant difference was identified as determined by unpaired two-tail t-test.

### Anti-ASP treatment results in increased energy expenditure and glucose oxidation

**Figure 3:**
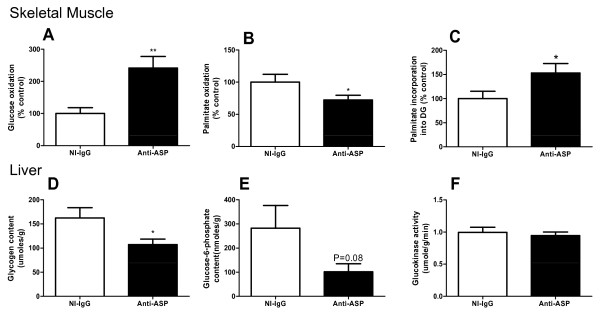
**Markers of skeletal muscle and liver energy expenditure and glucose storage**. Ex vivo quadriceps muscle (A) glucose oxidation, (B) fatty acid oxidation and (C) fatty acid incorporation into diglyceride. Ex vivo liver (D) glycogen content, (E) glucose-6-phosphate content and (F) glucokinase activity. All values are presented as mean ± SEM for NI-IgG treated mice (n = 6, white bars) and Anti-ASP treated mice (n = 7, black bars), where* P < 0.05 and ** P < 0.01 analyzed by unpaired two-tailed t-test.

Twenty-four hour energy expenditure was significantly elevated in Anti-ASP treated mice (P < 0.0001, Figure 2D). Analysis of dark and light 12-h cycles separately indicates a significant increase with anti-ASP for both time periods (P < 0.0001, 2-way ANOVA, AUC Figure 2E). While RQ increased with feeding (indicating increased glucose utilization during this period), there was no change in RQ in either dark or light cycles between the two groups, suggesting a whole body increase in both glucose and fatty acid oxidation (Figure 2F). To investigate the mechanism of augmented whole body energy expenditure, we evaluated glucose and fatty acid consumption in skeletal muscle and liver. In the skeletal muscle, glucose oxidation was significantly increased more than two-fold in Anti-ASP treated mice (P < 0.01, Figure 3A). On the other hand, palmitate oxidation was reduced (P < 0.05, Figure 3B) with a shift of palmitate from oxidation towards incorporation into diglycerides (P = 0.05, Figure 3C) with no change in TG incorporation or total TG mass (data not shown). In the liver, glycogen content was decreased by 34% (P < 0.05, Figure 3D) associated with a trend in reduced glucose-6-phosphate content (P = 0.08, Figure 3E) and no change in glucokinase (GK) activity (NS, Figure 3F). Also, there was no difference in liver TG mass (NI-IgG: 40.6 ± 4.6 μmoles/g and Anti-ASP: 43.0 ± 5.3 μmoles/g, NS, n = 5-7).

### NEFA levels were reduced with recombinant ASP treatment

**Figure 4:**
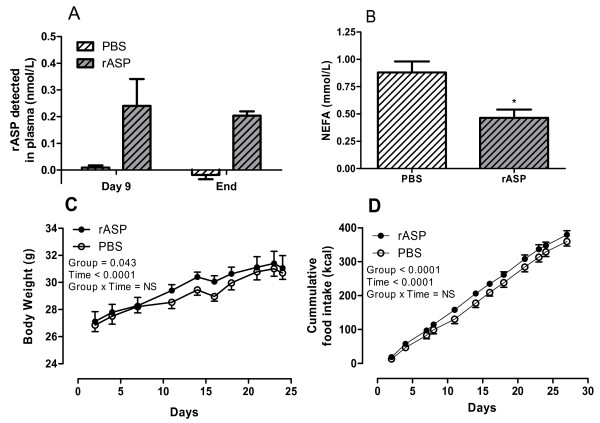
**Effects of recombinant ASP treatment on plasma NEFA levels, body weight and food intake in wildtype mice**. Fasting plasma samples from PBS treated mice (n = 6, white hatched bars) and rASP treated mice (n = 5, gray hatched bars). (A) Human rASP detected in mouse plasma following 9 days and 4 weeks treatment. (B) Fasting NEFA levels after 4 weeks treatment. Results were analyzed by unpaired two-tailed t-test. (C) Body weight (g) and (D) cumulative food intake (kcal) for PBS (n = 6, white circles) and rASP (n = 5, black circles) treated mice. Results were analyzed by two-way ANOVA. Group and Time P values are reported in graph. All values are presented as mean ± SEM, where * P < 0.05.

Next, we treated WT mice with human rASP for 4 weeks to enhance ASP action. Immunoreactive circulating human rASP was detected at ~0.3 pmol/mL on day 9 and remained constant until the end of the study period (Figure 4A). Interestingly, NEFA levels were significantly lower in the rASP treated mice (P < 0.05, Figure 3B). Fasting glucose levels were similar between rASP and PBS treated mice (PBS: 6.77 ± 0.74 and rASP: 6.81 ± 0.58 mmol/L, NS, n = 5-6). In addition, no difference in plasma TG, insulin or adiponectin were detected between PBS and rASP treated mice, however C3 levels were significantly elevated in the rASP treated mice (Table 2).

**Table 2:** PBS and rASP Treated Mice Plasma, Muscle and Liver Analysis

Plasma	PBS (n = 6)	rASP (n = 5)
Adiponectin (ug/mL)	8.48 ± 0.42	8.00 ± 0.51

Insulin (ng/mL)	0.34 ± 0.06	0.31 ± 0.08

IL-6 (pg/mL)	24.72 ± 0.88	23.65 ± 1.18

CRP (pg/mL)	1.51 ± 0.38	1.00 ± 0.16

C3 (mg/mL)	0.36 ± 0.02	0.48 ± 0.03*

Triglycerides (mmol/L)	0.99 ± 0.24	0.63 ± 0.04

Muscle		

Glucose Oxidation (% PBS)	100.0 ± 11.3	73.5 ± 12.6

Palmitate Oxidation (%PBS)	100.0 ± 8.8	96.4 ± 10.2

Liver		

Glycogen content (umoles/g)	142.3 ± 18.9	226.9 ± 31.2*

G6P content (nmoles/g)	221.6 ± 82.0	342.8 ± 57.1

Triglyceride mass (umoles/g)	34.2 ± 2.0	32.8 ± 3.0

GK activity (umoles/g/min)	0.69 ± 0.06	0.72 ± 0.07

Values are presented as mean ± SEM where * P < 0.05 as determined by unpaired two-tail t-test.

### rASP treatment increases body weight and food intake in WT mice

**Figure 5:**
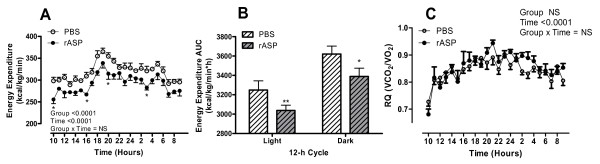
**Indirect calorimetry results for rASP treated mice**. Energy expenditure (A) and respiratory quotient (C) were monitored continuously over 24 hours for PBS (n = 6, white circles) and rASP (n = 5, black circles) treated mice. Results were analyzed by two-way ANOVA. Group and Time P values are reported in graph, their interactions were not significant. Analysis of energy expenditure in light and dark periods separately (2-way ANOVA) indicated a significant difference between Anti-ASP and NI-IgG for both time periods (both P < 0.0001), and the calculated area-under the curve (AUC) is shown in (B). All values are presented as mean ± SEM, where * P < 0.05, ** P < 0.01.

Monitoring of body weight during the treatment period indicated a significant increase in the rASP treated group within the middle time period of treatment (days 9-24, Figure 4C, P < 0.0043). However, at the end, a similar final fasting body weight was measured (PBS: 30.0 ± 0.6 g and rASP: 30.3 ± 0.8 g, NS, n = 5-6). This increase in body weight was associated with a small but significant increase in cumulative food intake (P < 0.0001, Figure 4D). Food efficiency, calculated as food intake relative to body weight, was comparable between the groups (PBS: 127.5 ± 18.8 kcal/g and rASP: 151.0 ± 41.2 kcal/g, NS). During this time period, energy expenditure was significantly reduced in rASP treated mice (P < 0.0001, Figure 5A), in both the light and dark cycles (Figure 5B). While respiratory quotient changes with feeding, there was no difference between the groups (Figure 5C). Analyses of ex vivo skeletal muscle oxidation revealed no change in glucose or palmitate oxidation for rASP and control mice (Table 2). Meanwhile, liver glycogen content was significantly increased by 59% in rASP treated mice (P < 0.05) with no change in glucose-6-phophate or TG content (Table 2).

## Discussion

The present study examines the effects of constant delivery of an ASP-neutralizing antibody or rASP in high-fat diet fed mice. Significant increases in energy expenditure and glucose oxidation coupled to reduced glycogen content were observed in Anti-ASP treated mice. In accordance with the Randle cycle [25], muscle fatty acid oxidation was suppressed while the glycolytic machinery was upregulated. On the other hand, rASP treated mice displayed reduced energy expenditure with increases in body weight (transient), and increased food intake and liver glycogen content. Therefore, Anti-ASP treatment increased whole body energy expenditure and glucose utilization in the muscle and decreased glucose storage in the liver while rASP treatment showed the opposing effects of decreased energy expenditure and increased liver glycogen.

Some of the limitations of the study include the small sample number and the reduced antibody levels detected towards the end of the study. While in cultured mouse adipocytes, low Anti-ASP antibody levels were sufficient to neutralize ASP-stimulated fatty acid uptake, the lack of body weight change between Anti-ASP and NI-IgG treated mice at study end could potentially be explained by reduced neutralization capacity due to the low circulating antibody levels detected. Therefore, in future studies, a longer lasting ASP blocking function, such as might be obtained with FAb fragments or antagonists, would be the ideal intervention tool.

Elevated fasting plasma ASP levels are often present in obesity and associated diseases and correlate with BMI as well as percent body fat (reviewed in [1]). Previous reports have shown that ASP stimulates fatty acid trapping in white adipose tissue [26] and adipocytes [7], stimulating fatty acid uptake and esterification in both preadipocytes and mature adipocytes [5,27]. In the present study, rASP treatment reduced fasting plasma NEFA levels, suggesting increased fatty acid uptake and storage. Further we showed that exogenous rASP stimulated weight gain and food intake while decreasing energy expenditure in mice. Consistent with these *in vivo *and *in vitro *effects, we hypothesize that ASP may play a role in increased weight gain.

In our previous pilot study, we demonstrated that Anti-ASP significantly increased muscle and liver AMPK activity as well as reducing muscle LPL activity [17]. While this was a short-term (10 days) daily injection study in low-fat diet fed mice, this study provided strong support for ongoing evaluation, and is consistent with our present study. In both the 10 day and 4 week studies, there was no change in food intake compared to control WT mice. In addition, Anti-ASP treatment in the two studies had no effect on metabolic hormones such as insulin or adiponectin levels. Further, our previous pilot study suggested an increase in energy expenditure via an increase in AMPK activity, a well known marker of a cell's "fuel gauge", indicating ATP/AMP availability [28].  We directly demonstrate that in our present study of Anti-ASP treated mice. Finally, Anti-ASP treatment does not promote liver lipotoxicity, as both studies showed no change in liver TG accumulation compared to controls.

As mentioned above, a significant outcome of blocking ASP in WT mice was increased whole body energy expenditure, evaluated by indirect calorimetery. Interestingly, C3KO mice on either a low-fat diet [15] or a high-fat diet [12] also displayed increased oxygen consumption, suggesting a role for ASP in energy metabolism. However while C3KO mice demonstrated a decrease in RQ (suggesting a preferential increase in fat oxidation), in the present Anti-ASP study, there was no change in RQ, suggesting a comparable increase in both fat and glucose oxidation. One major site of energy utilization is muscle. On a high fat diet, C3KO mice demonstrated greater fatty acid oxidation vs WT mice [12], while Anti-ASP mice demonstrate increased glucose oxidation vs WT mice in skeletal muscle. There are several factors that could explain this difference including length of time on a high-fat diet (12 weeks for C3KO mice, 6 weeks in the present study) or *in utero *compensatory mechanisms in C3KO mice. Either way, energy expenditure is increased. An additional point of difference between C3KO and Anti-ASP treated mice is their food intake. C3KO mice are hyperphagic [11,12], while there was no change in food intake in the present study. Again, differences could be related to time of treatment (4 weeks antibody injection vs in utero changes). Possibly, C3KO hyperphagia is centrally regulated as C5L2 (ASP receptor) has been identified in the brain [29]. We speculate that peripherally administered Anti-ASP treatment had no effect on food intake, because of a lack of central targeting, since antibodies cannot cross the blood brain barrier, although this remains to be evaluated. In summary, ASP neutralization effectively augmented energy expenditure without negatively affecting food intake.

It is interesting to note that the physiological results observed with either treatment are independent of changes in adiponectin, insulin and CRP levels, however Anti-ASP treated mice did display slightly increased plasma IL-6. IL-6 is a pro-inflammatory cytokine shown to influence glucose metabolism, however, its effects on insulin sensitivity remain controversial [30,31]. Wernstedt et al. showed that IL-6 KO mice had increased plasma ASP levels prior to the development of their mature onset obesity, and IL-6 injection rapidly reduced circulating ASP levels [32]. There appears to be a reciprocal relationship between IL-6 and ASP levels although the exact nature of this regulation is unknown and warrants further investigation.

## Conclusion

In summary, enhancing ASP action with the addition of recombinant ASP resulted in increased body weight and food intake, supporting a direct role of increased ASP in promoting obesity. Furthermore, targeting ASP decreased adipose tissue TG mass and LPL activity in our previous study [17] and increased energy expenditure, oxygen consumption and muscle glucose oxidation in the present study. Overall, Anti-ASP increased whole body energy utilization while rASP increases energy storage. Therefore, ASP may be a mediator of energy regulation. However, a direct role for ASP in tissues that express its receptor C5L2, such as muscle, liver and central nervous system [29], remains to be determined.

## List of Abbreviations

ASP: acylation stimulating protein; Anti-ASP: antibody against ASP; AMPK: AMP kinase; BMI: body mass index; C3: complement 3; CRP: C-reactive protein; DGAT: diaclyglycerol acyltransferase; G6P: glucose-6-phosphate; GK: glucokinase; IgG: immunoglobulin G; IL-6: interleukin-6; KO: knockout; LPL: lipoprotein lipase; NEFA: non-esterified fatty acid; NI: non-immune; PCOS: polycystic ovarian syndrome; rASP: recombinant ASP; RQ: respiratory quotient; TG: triglycerides; VCO_2_: volume of carbon dioxide production; VO_2_: volume of oxygen consumption; WT: wildtype.

## Authors' contributions

SP was involved in study design, mouse handling (body weight, food intake, indirect calorimetry, blood sampling), measured plasma hormones, performed palmitate oxidation assay, data analysis, and was the primary writer of the manuscript. AF performed the ex vivo glucose oxidation assay and measured liver substrate content, assisted with data analysis and manuscript editing. MM performed in vitro fatty acid uptake assay and enzymatic assays. YG measured plasma rASP and lipid levels. DR provided calorimetry equipment and was a study researcher. KC was a study researcher, assisted with study design and manuscript editing. All authors read and approved the final manuscript.

## Authors' Information

KC holds a Canada Research Chair in Adipose Tissue.
